# A Whole New Comprehension about ncRNA-Encoded Peptides/Proteins in Cancers

**DOI:** 10.3390/cancers14215196

**Published:** 2022-10-23

**Authors:** Qinnan Chen, Hongyu Shen, Fengqi Nie, Ming Sun

**Affiliations:** 1 Department of Clinical Medicine, Jiangsu Health Vocational College, 69 Huangshanling Rd., Nanjing 211800, China; 2 Department of Oncology Center, The Affiliated Suzhou Hospital of Nanjing Medical University, Suzhou Municipal Hospital, Gusu School, 458 Shizi St., Suzhou 215026, China; 3 Department of General Surgery, The First Affiliated Hosptal of Nanjing Medical University, 300 Guangzhou Rd., Nanjing 210003, China; 4 Department of Oncology, The Second Affiliated Hospital of Nanjing Medical University, 121 Jiangjiayuan Rd., Nanjing 210003, China

**Keywords:** peptides/proteins encoded by ncRNAs, translation mechanism, cancers, clinical applications

## Abstract

**Simple Summary:**

The advent of bioinformatics and high-throughput sequencing have disclosed the complexity of ORFs in ncRNAs. Thus, there is a dire need to deep into the real role of ncRNA-encoded proteins/peptides. Considerable progress has been achieved in several fields, ranging from the mechanism translation of ORFs in ncRNAs to various reliable detection means and experimental approaches. Several studies have been stressing functions and mechanisms of ncRNA-encoded peptides/proteins in cancers, which are helpful for us to understand the specific biological regulating procedure. Innovative research on animal models confirms the potential of clinical applications, such as being tumor biomarkers, antitumor drugs and cancer vaccines. In this review, we conclude the latest discoveries of ncRNA-encoded peptides/proteins, we are looking forwards to accelerating the pace of detection and diagnosis development in cancers.

**Abstract:**

It is generally considered that non-coding RNAs do not encode proteins; however, more recently, studies have shown that lncRNAs and circRNAs have ORFs which are regions that code for peptides/protein. On account of the lack of 5′cap structure, translation of circRNAs is driven by IRESs, m6A modification or through rolling amplification. An increasing body of evidence have revealed different functions and mechanisms of ncRNA-encoded peptides/proteins in cancers, including regulation of signal transduction (Wnt/β-catenin signaling, AKT-related signaling, MAPK signaling and other signaling), cellular metabolism (Glucose metabolism and Lipid metabolism), protein stability, transcriptional regulation, posttranscriptional regulation (regulation of RNA stability, mRNA splicing and translation initiation). In addition, we conclude the existing detection technologies and the potential of clinical applications in cancer therapy.

## 1. Introduction

In the past several years, numerous studies have shown that noncoding RNAs (ncRNAs) are involved in various biological activities, including chromosome modification and transcriptional and posttranscriptional regulation [1]. Due to their limited protein coding potential, it was once firmly recognized that ncRNAs didn’t function by encoding proteins [2]. To date, the open reading frames (ORFs) of all gene loci have not been entirely disclosed in the human genome, especially the small ORF (sORF), which contains less than 100 aa and has always been neglected. Hence, a range of RNA molecules are classified as ncRNAs. However, with the aid of proteomics and translation identification methods, we have realized that ncRNAs containing ORFs are translatable, including sORFs, even if they are small and undetectable [3].

**Figure 1:**
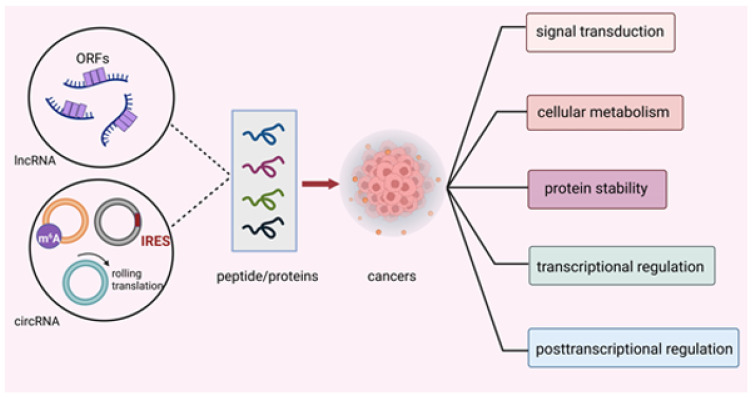
Translation mechanisms of ncRNA-encoded peptides/proteins. Peptides/proteins encoded by ncRNAs exhibited their effect through modulating signal transduction, cellular metabolism, protein stability, transcriptional and post-transcriptional activity in cancers.

For instance, Jackson et al. found that short and non-ATG-initiated ORFs in nonprotein coding genes could generate stable proteins in mice [4]. Wang et al. found that some long noncoding RNAs (lncRNAs) could bind to ribosomes through full-length mRNA analyses. This evidence suggests that lncRNAs have the capacity to produce proteins [5]. Later, Lu et al. detected 308 new proteins encoded by lncRNAs, one of which, UBAP1-AST6, was highly expressed in lung cancer cell lines, playing a critical role in cell proliferation. Chen et al. first stated that eukaryotic ribosomes could theoretically promote the translation of circRNAs by internal ribosome entry site (IRES) [6]. Subsequently, Nagarjuna et al. discovered an association between circRNAs and translating ribosomes and detected a protein encoded by circRNA generated from muscleblind by mass spectrometry (MS) [7]. These findings indicated that ncRNAs yielded proteins or peptides that could serve as important regulators instead of transcriptional noise in biological activities [8]. Since we have a new understanding of ncRNAs, it is necessary for us to unveil the mysterious roles ncRNA-derived peptides/proteins play in cellular activities. In this review, we summarize the mechanisms of translation initiation in ncRNA-encoded peptides/proteins and elucidate the vital potential of peptides/proteins encoded by ncRNAs in cancers (Figure 1) and the possibility of their application in clinical remedies.

## 2. Translation of ORFs in ncRNA

### 2.1. Translation of ORFs in lncRNAs

Structurally, ORF is a region containing nucleotide sequence in the context of mRNAs and other RNAs or transcripts that can be translated from the start codon to the stop codon. The ribosome starts translation from the start codon and elongate peptides chain along mRNA sequence. When ribosome encounters the stop codon, the elongation terminates. LncRNAs are the transcripts with mRNA-like feature: capping and polyadenylation, but they were not thought to be translatable. ORFs are classified into two big groups: canonical ORFs and noncanonical ORFs (nORFs). In nORFs, there five different types: intergenic ORFs, upstream ORFs (uORFs), long noncoding ORFs (lncORFs), short coding sequences (short CDSs), and short isoform ORFs. Studies have verified nORFs can be translated in to functional, noncanonical peptides. Moreover, translation of lncORFs is mostly indistinguishable from that of ORFs in mRNA, and a strong preference for lncORF translation often initiates at the AUG start codons. Moreover, a recent report pointed out that mRNAs containing short CDSs are one subgroup of annotated lncRNAs [9,10,11]. For example, Chen et al. found that short lncRNA CDSs could encode functional micropeptides [12].

### 2.2. Translation of ORFs in circRNAs

CircRNAs are generated by a special process known as backsplicing that links the 5′ and 3′ ends of exons and/or introns. Though circRNAs lack 5′ cap structure, the translation can be initiated by IRES element, m6A modification or through rolling amplication [13]. The translation of both poliovirus and encephalomyocarditis virus RNA is induced by IRESs. While IRESs have been discovered in an increasing number of eukaryotic mRNAs, they have been identified as crucial RNA scaffolds recruiting ribosomes with the assistance of IRES-transacting factors (ITAFs) for translation initiation [14]. For example, circ-E-cad RNA junction reads were detected in glioblastoma (GBM) samples via ribosome profiling, an IRES was identified, and its activity was measured. Thus, the product circRNA-encoded E-cadherin could be efficiently translated from circ-E-cad [15]. Dramatically, Chen et al. found elements of the special circular IRES structure that could facilitate circular IRES translation activity: a higher GC content and lower minimum free energy (MFE). Further investigation showed two pivotal regulators in which the 18S rRNA complementary sequence and 40–60 nt stem-loop structured RNA element (SuRE) on the IRES promoted endogenous circRNA translation [16].

Another cap-independent translation mechanism relies on m^6^A modification. For instance, Yang et al. found that the insertion of an IRES sequence into circRNAs could initiate translation; however, the protein was also encoded in the control group without an IRES but contained m^6^A sites. The results suggested that m^6^A modification could drive protein translation, which was further verified in several studies [17,18]. Since m^6^A modification is enriched in circRNAs, it could be regarded as an “IRES”. Further experiments concluded that the m^6^A reader YTH domain family protein 3 (YTHD3) could recruit the translation initiation factor eIF4G2, and the translation procedure was promoted by the adenosine methyltransferase METTL3/14. Moreover, the m^6^A-mediated translation of endogenous circRNAs was predicted to be induced by heat shock [19]. Consistently, it has been reported that the E7 oncoprotein is generated from HPV-derived circE7 via m^6^A-dependent translation in a heat shock manner [17,20]. Traditionally, in linear RNA, when the ribosome encounters a stop codon, it dissociates from the RNA, and then the ribosome enters the initiation-elongation-termination process again. In circRNAs containing an infinite ORF, the ribosome circles the RNA template following an initiation process, leading to the production of a long repeating peptide. This translation manner is similar to rolling circle amplification, an isothermal and enzymatic procedure modulated by DNA polymerases, and it has been revealed in both prokaryotic and eukaryotic systems [21,22,23]. Interestingly, Liu et al. reported that circEGFR could produce a functional and endogenous ‘rolling-translated’ product termed rtEGFR; although there was no in-frame stop codon, translation of circEGFR could be terminated by a specific out-of-frame stop codon [24]. This study provides a new understanding of the rolling translation of circRNA.

## 3. Functions and Mechanisms of ncRNA-Encoded Peptides/Proteins in Cancers

Peptides/proteins encoded by ncRNAs have been confirmed to be related to multiple bio logical and pathophysiological processes, including muscle regeneration [25], metabolism [26], embryonic development [27], inflammation and immunity [28]. In addition, emerging evidence suggests that these peptides/proteins are considerable players in tumor progression. Here, we will expand upon their roles according to different functions and mechanisms.

### 3.1. Regulation of Signal Transduction

#### 3.1.1. Wnt/β-Catenin Signaling

**Figure 2:**
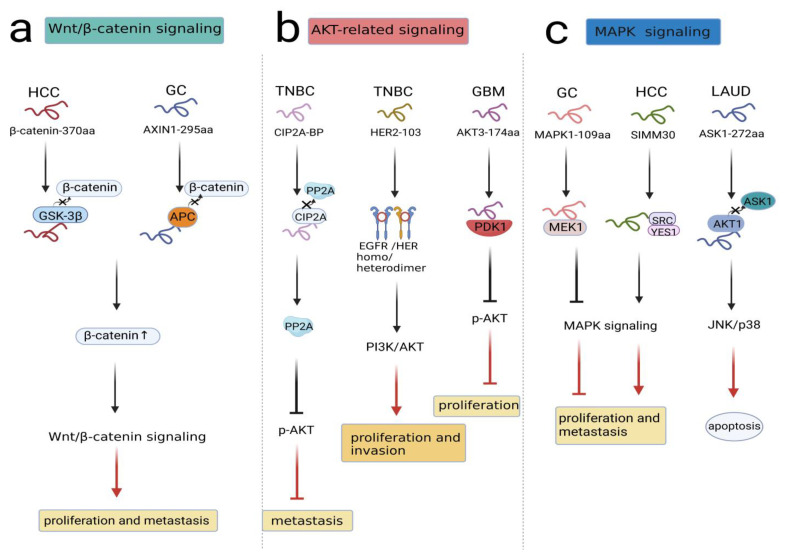
NcRNA-encoded peptides/proteins regulated signal transduction. (**a**) β-Catenin-370aa and AXIN1-295aa competitively bound to component(GSK-3β, APC) in destruction complex to activate Wnt/β-catenin pathway, promoting proliferation and metastasis in HCC and GC. (**b**) CIP2A-BP competitively bound to CIP2A, prohibiting p-AKT and TNBC metastasis; HER2-103 induced EGFR/HER3 homo/heterodimer formation and downregulated PI3K/AKT activation, promoting proliferation and invasion in TNBC; AKT3-174aa combine with PDK1 to stimulate p-AKT activation, prohibiting proliferation in GBM. (**c**) MAPK1-109aa and SMIM30 interacted with MEK1, SRC/YES1 to regulate MAPK signaling, influencing proliferation and metastasis in GC and HCC; ASK1-272aa competitively boundto AKT1 to activate ASK1/JNK/p38 signaling, promoting apoptosis in LAUD.

As a highly conserved pathway, the Wnt/β-catenin pathway is widely involved in a number of malignant events, such as cell proliferation, cell apoptosis, vasculogenesis and metastasis [29,30,31,32]. In general, β-catenin is located in the cytoplasm and degraded by the destruction complex, which consists of Axin, adenomatosis polyposis coli (APC), glycogen synthase kinase 3β (GSK-3β), protein phosphatase 2A (PP2A), and casein kinase 1α (CK1α) in the absence of Wnt ligand. However, when the Wnt ligand combines with FZD and LRP5/6, β-catenin degradation can be inhibited, and it translocates from the cytoplasm to the nucleus, where β-catenin is recruited with TCF/LEF to form a transcriptional complex regulating target gene expression [33,34]. A recent study revealed circβ-catenin as an oncogenic circRNA related to the Wnt/β-catenin pathway in hepatocellular carcinoma(HCC). circβ-catenin knockdown suppressed the Wnt/β-catenin pathway. The circβ-catenin translation product was termed β-catenin-370aa, which competitively binded with GSK-3β; in this case, endogenous β-catenin was not degraded, and the Wnt/β-catenin pathway was activated. Therefore, circβ-catenin encoding β-catenin-370aa promoted the proliferation and metastasis of HCC via the Wnt/β-catenin pathway [35]. Similarly, based on circRNA sequencing data, circAXIN1 was found to be highly expressed in gastric cancer (GC), and it encoded the protein AXIN1-295aa. As a competitor, AXIN1-295aa interacted with APC, leading to the inability to form the destruction complex. Subsequently, β-catenin accumulated in the nucleus, activating downstream genes and inducing tumorigenesis and progression [36].(Figure 2a).

In conclusion, these findings suggested that peptides/proteins encoded by ncRNAs combine with the destruction complex to activate Wnt/β-catenin signaling in cancer cells.

#### 3.1.2. AKT-Related Signaling

Numerous studies have revealed that AKT plays a key role in driving different signaling pathways that contribute to cancer growth. Downregulation of certain tumor suppressors or upregulation of oncogenes can lead to initiation of the AKT-related signaling pathway [37]. For instance, LINC00665 encoded a novel micropeptide, CIP2A-BP, which was downregulated in triple-negative breast cancer (TNBC) cells. Simultaneously, PP2A functioned as a tumor inhibitor, while CIP2A served as an oncogene. CIP2A-BP competed with PP2A subunit B56γ to bind to CIP2A, releasing PP2A and prohibiting phosphorylation of AKT. Therefore, the expression of MMP2, MMP9, and Snail was decreased, and TNBC metastasis was prevented [38]. In addition, HER2-103 encoded by circ-HER2 could induce EGFR/HER3 homo/heterodimer formation and downregulate PI3K/AKT activation to maintain malignant phenotypes in TNBC. In contrast, HER2-103 silencing reduced p-EGFR and p-AKT levels, indicating the essential existence of HER2-103 in the signaling network [39]. Moreover, circ-AKT3 encoded a novel protein named AKT3-174aa, which was expressed at low levels in GBM. AKT thr308 was easily exposed to p-PDK1 due to the low expression of AKT3-174aa, promoting AKT activation cascades to decrease tumor proliferation and reverse radiation resistance [40] (Figure 2b). In summary, these findings indicated that peptides/proteins encoded by ncRNAs influenced malignant behaviors through AKT-related signal transduction.

#### 3.1.3. MAPK Signaling

Mitogen-activated protein kinase (MAPK) signaling is a crucial inducer in human cancers. At least three MAPK signaling module functions have been characterized in mammalian cells: ERK, JNK/SAPK and p38 MAPK [41]. The MAPK/ERK pathway, also known as the Ras-Raf-MEK-ERK pathway, is a conservative pathway in tumor progression. When extracellular stimulating factors bind to transmembrane receptors, the intracellular response can be triggered. Under these circumstances, Ras activation activates the protein kinase activity of RAF kinase composed of A-Raf, B-Raf, and C-Raf, and then the phosphorylation cascade of RAF-MEK1/2- ERK1/2-MAPK1/2 occurs, leading to the hyperactivation of the MAPK signaling pathway [42,43]. Several ncRNA-peptides/proteins have been found to function in the MAPK signaling pathway. circMAPK1 suppressed tumorigenesis and metastasis in GC, and its translational product was MAPK1-109aa. Although MAPK1-109aa shared most of its sequence with MAPK1, MAPK1-109aa exerted an entirely opposite function compared to MAPK1. MAPK1-109aa competitively interacted with MEK1 to inhibit MAPK1 phosphorylation, negatively regulating the proliferative and invasive behavior of gastric cancer cells [44]. Recently, SMIM30 was shown to be encoded by LINC00998 and promoted proliferation and migration in HCC. Remarkably, SMIM30 was proven to be a membrane peptide that could anchor and activate SRC/YES1, which were essential mediators of signaling pathways in cancers. Then, the SRC/YES1-SMIM30 complex could activate MAPK signaling and promote HCC development [45].

Additionally, apoptosis signal-regulating kinase 1 (ASK1) is a member of the MAP3K family, which is the main component in MAPK signaling. Various injury stressors and inflammatory factors can activate ASK1, which results in the activation of the JNK and p38 MAPK pathways, thus promoting cell death [46]. Specifically, ASK1 has been illustrated as a modulator in chemotherapy-mediated cell apoptosis [47]. Wang et al. detected a circASK1-encoded isoform named ASK1-272aa in lung adenocarcinoma (LUAD) cells. ASK1-272aa regulated the inhibitory effect of its host gene on gefitinib resistance. Mechanistically, ASK1-272aa suppressed ASK1 phosphorylation at the distinct site S83 by competitively recruiting AKT1, thereby restoring the proapoptotic effect of ASK1/JNK/p38 signaling and augmenting gefitinib sensitivity in LAUD cells [48] (Figure 2c). Collectively, these studies documented that peptides/proteins encoded by ncRNAs were essential players in MAPK signaling.

#### 3.1.4. Other Signaling

A previous study revealed that 9% of HBV-related HCC cases are linked to the abnormal activation of JAK/STAT. The JAK/STAT pathway can be activated by various ligands, such as cytokines and growth factors [49]. The HBVPTPAP peptide encoded by the lncRNA HBVPTPAP could interact with the transmembrane receptor PILRA, which negatively regulated JAK/STAT signaling, resulting in the promotion of cell apoptosis in HCC [50]. Hedgehog (Hh) signaling is involved in various neurological disorders, and the most studied Hh ligand is sonic hedgehog (Shh) [51,52]. Wu et al. found that the oncogenic protein SMO-193aa generated from circ-SMO maintained CSC self-renewal ability and tumorigenicity in GBM. As a central player, SMO-193aa was essential in Hh signaling transduction. It could increase SMO cholesterol modification and derepress SMO from the protein receptor Ptch1 upon Shh stimulation, resulting in the activation of the signaling pathway. Moreover, SMO-193aa was positively modulated by FUS, a transcriptional target of Gli1 in Hh signaling [53]. YAP1 acts as a transcriptional coactivator in the Hippo signaling pathway, and Hippo-YAP signaling promotes malignant processes for cell cycle progression, epithelial-mesenchymal transition (EMT), cell motility, and metastasis [54]. CircPPP1R12A-73aa encoded by circPPP1R12A contributed to proliferation and metastasis in colon cancer (CC). In contrast, in YAP1-silenced CC cells, the promotive effect of circPPP1R12A-73aa was obviously alleviated [55]. Gu et al. identified a novel circGprc5a peptide produced by circGprc5a via an autocrine pathway in bladder cancer. Furthermore, they found that circGprc5a-peptide combined with Gprc5a membrane protein to launch G-protein-coupled receptor (GPCR) signaling, thus driving bladder CSC self-renewal and metastasis [56]. On the whole, multiple lines of evidence have shown that peptides/proteins encoded by ncRNAs affected carcinogenesis via different signaling pathways, and research on these signal transduction pathways allowed us to detect new strategies in cancers.

### 3.2. Regulation of Cellular Metabolism

#### 3.2.1. Glucose Metabolism

Cellular metabolism requires the consumption of particular nutrients, among which glucose, fatty acids and amino acids are the principal components involved in biosynthetic reactions. Unlike normal cells, tumor cells are “hungry”, and they are considered to seize nutrients to sustain rapid growth potential via metabolic reprogramming, which is accompanied by alterations in gene levels, cell differentiation and the cancer microenvironment [57,58,59,60]. In normal cells, pyruvate formed by glycolysis of glucose enters mitochondria, where it is oxidized through the tricarboxylic acid cycle (TAC) and generates ATP to satisfy the energy needs of the cell. However, in cancer cells, most pyruvate is deoxygenated to lactic acid instead of entering the mitochondria. Lactic acid is typically produced in the absence of oxygen, but even when oxygen is abundant, cancer cells preferentially metabolize glucose into lactic acid. This process is known as “aerobic glycolysis” or the Warburg effect. The Warburg effect exacerbates the formation of acidic microenvironments, leading to aggressive malignant progression in human cancers [61].

**Figure 3:**
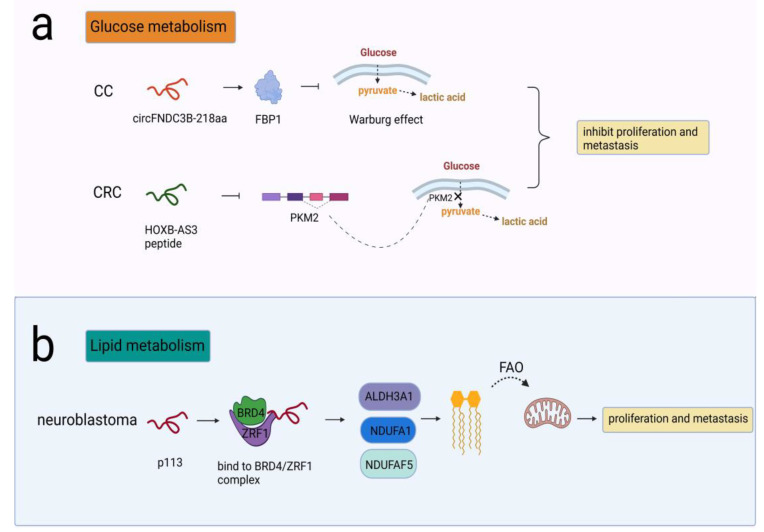
NcRNA-encoded peptides/proteins regulated cellular metabolism. (**a**) CircFNDC3B-218aa enhanced FBP1 and alleviated the Warburg effect, prohibiting proliferation and metastasis in CC; HOXB-AS3 peptide suppressed PKM splicing and PKM2 formation that was critical to the reprogramming of glucose metabolism, prohibiting proliferation and metastasis in CRC. (**b**) p113 combined with ZRF1/BRD4 to activate ALDH3A1, NDUFA1, and NDUFAF5 that were needed in conversion of fatty aldehydes into FAO, promoting proliferation and metastasis in neuroblastoma.

Some peptides/proteins have been reported to be involved in glucose metabolism to impact glycolysis. One example was circFNDC3B-218aa, encoded by circFNDC3B, which participated in the carcinogenesis of CC. CircFNDC3B-218aa inhibited proliferation and EMT progression by enhancing FBP1, which alleviated the Warburg effect by driving metabolic reprogramming from glycolysis to oxidative phosphorylation [62]. The other peptide was HOXB-AS3, which was translated from lncRNA HOXB-AS3 and exerted a tumor-suppressive effect in colorectal cancer (CRC). PKM2 was an isoform of the PK enzyme in the last step of glycolysis. The HOXB-AS3 peptide suppressed hnRNP A1-mediated PKM splicing and PKM2 formation, therefore inhibiting the reprogramming of glucose metabolism [63] (Figure 3a).

#### 3.2.2. Lipid Metabolism

Cancer cells also harness lipid metabolism to obtain the energy needed for their proliferation and metastasis. The typical changes in lipid metabolism include lipid uptake, synthesis and lipolysis, such as fatty acid β-oxidation (FAO). FAO is critical for ATP production, mitochondrial function and cell survival. Different types of tumors, such as glioma, TNBC and acute myeloid leukemia (AML), exhibit high FAO activity [64]. CUX1 circular RNA encoded a novel 113-amino acid protein p113 that combined with ZRF1/BRD4 to form a transcriptional complex. The complex induced transcriptional activation of ALDH3A1, NDUFA1, and NDUFAF5, thereby promoting the conversion of fatty aldehydes into FAO and enhancing mitochondrial activity in neuroblastoma cells [65] (Figure 3b).

Conclusively, these discoveries favored the viewpoint that peptides/proteins encoded by ncRNAs were critical events in tumor metabolic reprogramming and uncovered complex regulatory networks in cellular metabolism.

### 3.3. Regulation of Protein Stability by ncRNAs Yields Proteins/Peptides

There are two main proteolytic systems responsible for protein degradation in eukaryotic cells: the ubiquitin proteasome system (UPS) and the lysosomal system [66]. The UPS is involved in many cellular processes, such as ER stress [67], cell proliferation [68], and DNA damage recognition [69]. The UPS controls the degradation of substrates through the action of specific enzymes (E1, E2, E3 enzymes). Tumorigenesis progresses due to the deubiquitination of oncoproteins (such as Myc proteins, CycE, Notch1) [70,71]. Several peptides/proteins encoded by ncRNAs have been shown to participate in the UPS during cancer development. Yang et al. identified FBXW7-185aa in glioma cells. FBXW185aa inhibited cell proliferation and promoted cell cycle arrest by suppressing c-Myc. FBXW7-185aa interacted with the deubiquitinating enzyme USP28 and competitively released FBXW7α from USP28, thereby antagonizing the USP28-induced deubiquitination of c-Myc and sequentially destabilizing c-Myc [72]. Similarly, FBXW7-185aa upregulated FBXW7 expression and facilitated c-Myc degradation, thus reducing TNBC cell proliferation and metastasis [73]. In addition, Zhang et al. reported that the novel protein SHPRH146aa encoded by circ-SHPRH acted as an inhibitor in GBM. Microarray analysis revealed that SHPRH146aa was involved in the protein ubiquitination pathway. Notably, stabilized SHPRH acted as an E3 ligase that could degrade proliferation cell nuclear antigen (PCNA), and the E3 ligase DTL, could target SHPRH. SHPRH-146aa served as a guardian protecting full-length SHPRH from DTL-induced ubiquitination, which in turn promoted PCNA degradation [74,75].

**Figure 4:**
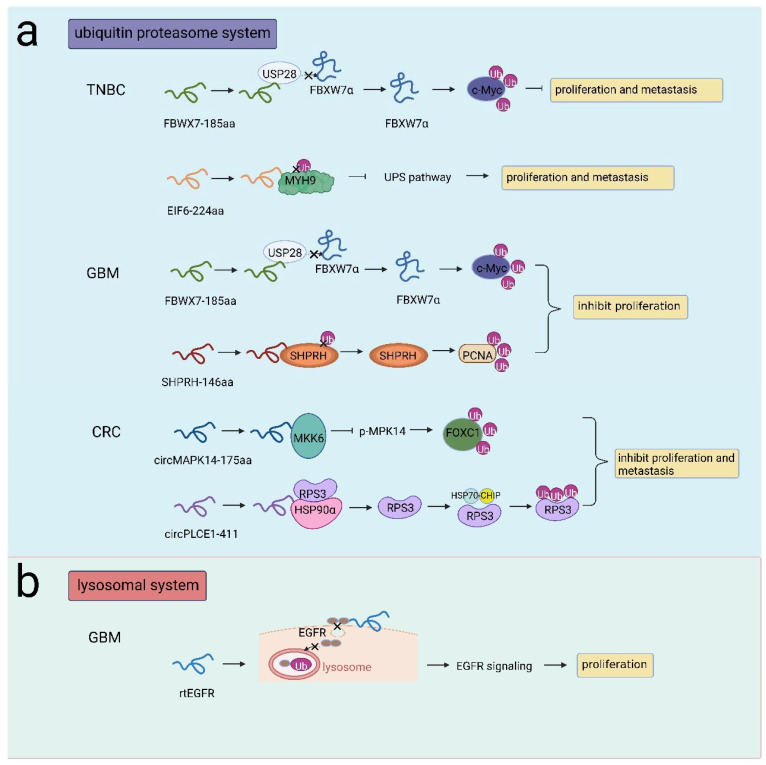
NcRNA-encoded peptides/proteins regulated protein stability. (**a**) FBXW7-185aa competitively interacted with USP28 to release FBXW7α and facilitated c-Myc degradation, inhibiting proliferation and metastasis in TNBC or inhibiting proliferation in GBM; EIF6-224 aa interacted with MYH9 to prohibited MYH9 degradation, promoting proliferation and metastasis in TNBC; SHPRH-146aa protected SHPRH from ubiquitination and induced PCNA degradation, promoting proliferation in GBM; circMAPK14-175aa competitively bound to MKK6 to repress p-MAPK14 and facilitated FOXC1 degradation, inhibiting proliferation and metastasis in CRC; circPLCE1-411 interacted with HSP90α/RPS3 to induce RPS3 dissociation, RPS3 interacted with HSP70-CHIP to induce RPS3 degradation, inhibiting proliferation and metastasis in CRC. (**b**) rtEGFR interacted with EGFR to reduce EGFR endocytosis and decrease EGFR ubiquitination in lysosomes, promoting proliferation in GBM.

In addition, EIF6-224 aa encoded by circ-EIF6 promoted the progression of TNBC cells. EIF6-224 aa could directly interact with the oncoprotein MYH9 and decreased the ubiquitination of MYH9 protein and prohibited MYH9 proteasomal degradation, therefore activating the Wnt/beta-catenin pathway and inducing TNBC proliferation and metastasis [76]. circMAPK14 inhibited CRC cell proliferation and metastasis by encoding the peptide circMAPK14-175aa. circMAPK14-175aa competitively bound to MKK6 to repress MAPK14 phosphorylation. As a result, nuclear translocation of MAPK14 was reduced, and FOXC1 was degraded via the UPS, which altered the expression of downstream genes related to the malignant phenotype in CRC [77]. Another similar functional protein in CRC was circPLCE1-411, which was encoded by circPLCE1 and acted as a tumor suppressor. circPLCE1-411 could bind to the HSP90α/RPS3 complex, inducing RPS3 dissociation. RPS3 was an NF-kB regulator that reduces the activation of NF-kB signaling in cell proliferation and metastasis. RPS3 interacted with the E3 ligase complex HSP70-CHIP, leading to the ubiquitin-dependent degradation of RPS3 [78] (Figure 4a).

As a membrane protein, EGFR can be activated by distinct ligands. Activated EGFR is dynamically recycled to the membrane or transported to lysosomes for degradation via endocytosis [79]. Abnormal activation of EGFR occurred in more than half of GBM cases. Liu et al. identified circEGFR, which was highly expressed in GBM, and its protein product was rtEGFR. rtEGFR was found to be localized in the cell membrane, on which rtEGFR directly interacted with EGFR, reducing EGFR endocytosis and decreasing EGFR ubiquitination in lysosomes. Therefore, EGFR signaling was aberrantly activated, and tumor progression was promoted [25] (Figure 4b). Herein, these studies focused on the regulation of protein stability by ncRNAs that yield proteins/peptides mainly through the UPS or lysosomal system, providing promising therapeutic targets in cancer treatments.

### 3.4. Transcriptional Regulation

**Figure 5:**
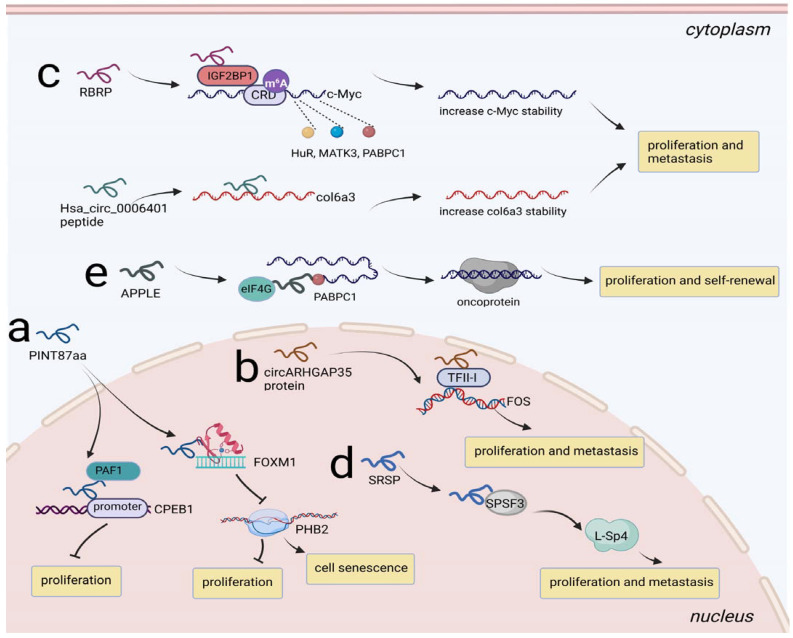
Regulation of NcRNA-encoded peptides/proteins at transcription and posttranscription level. (**a**) PINT87aa bound to FOXM1 to inhibit PHB2 transcription, promoting cell senescence and prohibiting proliferation in HCC; PINT87aa recruited PAF1 to CPEB1 promoter, limiting the transcriptional elongation of CPEB1 and prohibiting proliferation in GBM. (**b**) circARHGAP35 protein interacted with TFII-I and upregulated the levels of downstream gene FOS, promoting proliferation and metastasis in HCC. (**c**) RBRP enhanced the recruitment of IGF2BP1 to the m6A-modified mRNA CRD of c-Myc and strengthened the binding of HuR, MATK3, PABPC1 to c-Myc, stabilizing c-Myc and promoting proliferation and metastasis in CRC; Hsa_circ_0006401 peptide served as an RBP to decrease col6a3 mRNA decay, promoting proliferation and metastasis in CRC. (**d**) SRSP strengthened the recognition and interaction of SRSF3 to induced L-Sp4 formation, promoting proliferation and metastasis in CRC. (**e**) APPLE facilitated PABPC1-eIF4G interaction to induce oncoprotein synthesis, promoting proliferation and self-renewal in AML.

Dysregulation of transcription can cause a broad range of diseases, including cancers. Several peptides/proteins derived from ncRNAs have been shown to be involved in eukaryotic transcription, and there are some ways peptides/proteins can regulate transcription: by acting as decoys and inhibiting the binding of a transcriptional regulatory factor or recruiting a regulatory protein complex to a target gene [80]. Accordingly, we paid close attention to biological mechanisms mediated by ncRNA-encoded peptides/proteins at the transcriptional level. For instance, in HCC, circPINT was characterized as a translatable ncRNA that could encode PINT87aa, which induced cell proliferation and inhibited cell senescence. PINT87aa functioned by binding to the DNA-binding domain of FOXM1 and blocked FOXM1-mediated PHB2 transcription [81]. Coincidentally, PINT87aa served as a tumor suppressor in GBM. PINT87aa functioned as an anchor and recruited the PAF1 complex to the target gene CPEB1 promoter, subsequently limiting the transcriptional elongation of CPEB1 [82] (Figure 5a). In addition, circARHGAP35 protein derived from circARHGAP35 exerted its role as an oncogenic protein in HCC. Mechanistically, circARHGAP35 protein formed a complex with transcriptional regulator TFII-I and upregulated the levels of downstream gene FOS [18] (Figure 5b).

### 3.5. Posttranscriptional Regulation

#### 3.5.1. Regulation of RNA Stability

Mountain studies have emphasized RNA-binding proteins (RBPs) are critical in regulating RNA stability. RBP-mediated m^6^A modification can cause diverse tumors. m^6^A can guide molecular processes through m^6^A recognition of reader proteins. There are two kinds of m^6^A recognition reader proteins: YTHDF2 is involved in the decay of m^6^A-containing RNA, while insulin-like growth factor 2 mRNA-binding protein (IGF2BP) contributes to the opposite effect [83,84,85].Two peptides have been revealed to play roles in RNA stability regulation and have attracted much attention, including RNA-binding regulatory peptide (RBRP) and Hsa_circ_0006401 peptide. RBRP was encoded by LINC00226-1 and was upregulated in highly metastatic cancer cells and primary CRC tissues. RBRP enhanced the recruitment of IGF2BP1 to the m^6^A-modified mRNA CRD of c-Myc, thus promoting c-Myc mRNA recognition by IGF2BP1. Moreover, RBRP strengthened the binding of other RNA stabilizers, such as HuR, MATK3, PABPC1, to c-Myc in a m^6^A-dependent manner, further stabilizing c-Myc expression and thereby promoting tumorigenesis [86]. In addition, the Hsa_circ_0006401 peptide generated from circular RNA hsa_circ_0006401 promoted CRC metastasis by acting as an RBP to decrease col6a3 mRNA decay [87] (Figure 5c).

#### 3.5.2. Regulation of mRNA Splicing

Alternative splicing is one of the most prevalent posttranscriptional regulatory mechanisms contributing to proteomic diversity in eukaryotic cells. There are some typical abnormal splicing modes in tumors, such as exon inclusion or skipping, constitutive splicing, alternative 5′ or 3′ splice sites, intron retention and mutually exclusive exons [88]. Dysregulation of splicing factors leads to the occurrence of several human cancers. Recently, Meng et al. unveiled a protein termed splicing regulatory small protein (SRSP) derived from lncRNA LOC90024. In normal cells, SRSP was expressed at low levels so that the splicing factor SRSF3 could not recognize exon 3 of Sp4 well, resulting in exon 3 skipping and the formation of the splicing variant S-Sp4, which was noncancerous. In contrast, SRSP was highly expressed in CRC cells, and it strengthened the recognition and interaction of SRSF3 on exon 3 of Sp4 to promote exon 3 inclusion, which induced cancerous splicing variant L-Sp4 formation, eventually accelerating the pace of cancer progression [89] (Figure 5d).

#### 3.5.3. Regulation of Translation Initiation

Translation involves three steps: initiation, extension, and termination, while most mRNA translation regulation occurs in the initiation step. Some translation-associated proteins, including eIF and polyadenylate-binding proteins (PABPs), are required for the circularization and activation of mRNAs. Inaccurate mediation of these processes results in aberrant translation initiation, which satisfies the demands of oncogenes that need high protein synthesis in cancer [90,91]. An oncomicropeptide, APPLE, encoded by ASH1L-AS1, served as a translation initiation regulator in AML. It facilitated PABPC1-eIF4G interaction to induce mRNA circularization and eIF4F complex assembly, and in turn, the PABPC1-APPLE-eIF4G axis met the requirement of oncoprotein synthesis [92] (Figure 5e).

Overall, peptides/proteins encoded by ncRNAs acted as potential biomarkers and therapeutic targets by regulating RNA stability, mRNA splicing and translation initiation at the posttranscriptional level, these reports have expanded our knowledge about the functions of peptides/proteins encoded by ncRNAs in cancers.

### 3.6. Others

#### 3.6.1. Angiogenesis Inhibition

Angiogenesis is critical for tumor growth, which requires blood vessels for nutrients and oxygen. Vascular endothelial growth factor (VEGF) is a 40-kDa heterodimeric glycoprotein that plays a major role in pathological angiogenesis, stimulating endothelial cell proliferation, migration, and invasion [93,94]. Growing evidence has suggested that VEGF inhibition has particular antitumor effects [95]. Hsa-circ-0000437 encoded a functional peptide termed CORO1C-47aa. CORO1C-47aa negatively regulated endometrial tumor progression by competing with the transcription factor TACC3 for recruitment to ARNT, thereby inhibiting VEGF expression and reducing angiogenesis [96]. In addition, the polypeptide ASRPS translated from LINC00908 was found to be downregulated in TNBC. It directly bound to the coiled coil domain (CCD) domain of STAT3 and suppressed STAT3 activation, thus blocking STAT3/VEGF signaling and impeding angiogenesis [97].

#### 3.6.2. DNA Repair

DNA repair contributes to genomic alterations in tumor initiation and progression. A large amount of evidence suggests that poly(ADP-ribose) polymerase-1 (PARP-1) functions as a DNA damage detector. Once single-strand breaks (SSBs) and double-strand breaks (DSBs) occur, PARP-1 can quickly recognize and participate in the DNA damage response [98,99]. A novel DIDO1-529 aa protein, the translation product of CircDIDO1, could increase DNA damage in GC cells. Simultaneously, DIDO1-529aa interacted with PARP1 to inhibit the binding of PARP1 to damaged DNA and the enzymatic activity of PARP1. As a result, DIDO1-529aa exerted tumor suppressive roles by inducing GC cell apoptosis [100].

#### 3.6.3. Endoplasmic Reticulum (ER) Stress

The ER is responsible for correctly folding polypeptide chains and processing them into functional proteins. Some exogenous or endogenous factors lead to the accumulation of misfolded or unfolded proteins in the ER, which is called ER stress. In the presence of high levels of ER stress, the unfolded protein response will induce cells to commit self-destruction [101,102]. A small protein of 79 amino acids, FOXA1-regulated conserved small protein (FORCP), generated from LINC00675, functioned as an inhibitor in well-differentiated CRC cells. FORCP was primarily located in the ER and interacted with BRI3BP in response to ER stress, thus promoting CRC cell apoptosis [103].

#### 3.6.4. Immune Surveillance

Immune surveillance is one of the basic functions of the immune system. When tumorous gene mutation occurs, new antigenic determinants appear on the cell surface, and cytotoxic lymphocytes can recognize, kill and remove mutated cells in time to prevent the occurrence of tumors [104]. Yasuhiro et al. found that a tumor antigen named the PVT1 peptide was encoded by the lncRNA PVT1 and overexpressed in CRC tissues. The PVT1 peptide was identified by CD8+ T cells and could be presented by HLA class I, which implied the involvement of the PVT1 peptide in patient immune surveillance [105].

Therefore, peptides/proteins encoded by ncRNAs exert their effects by angiogenesis inhibition, DNA repair modulation, ER stress response and immune surveillance. In summary, current research has revealed that peptides/proteins translated from ncRNAs possess vital regulatory potential in tumors. It is of great significance to clarify the functions of ncRNA-encoded peptides/proteins in protein synthesis, and all these results have inspired us to search for more fundamental molecular mechanisms.

## 4. Future Prospects

In recent years, the findings of translatable ncRNAs have gained great attention in the life sciences. We have summarized the main translation mechanisms of peptides/proteins encoded by ncRNAs and their roles in cancer regulation. These peptides/proteins have exhibited powerful functions both in vivo and in vitro. At present, the discovery of peptides/proteins from ncRNAs is still at the initial stage, and a large number of them are still waiting to be found. Technologies based on translational mechanisms are constantly emerging and being updated, and the specific clinical applications of peptides in tumors remain to be explored.

### 4.1. Outlook for Technology

Currently, there are many methods and tools to determine the protein coding potential of ncRNAs. Bioinformatics techniques can predict ORFs in noncoding regions, and software commonly used to predict ORFs includes ORF Finder, CircRNADb, ORF score PhyloCSF, etc. Websites like nucleotide- and protein-protein BLAST and UCSC can be used for conservation analysis. Due to the different translation initial elements, there are alternative prediction methods for IRES and m^6^A modification [106]. In addition, ribosome-associated technologies have been extensively used in current research: ribosome profiling can recognize small ORFs, but this approach has a high false-positive rate [107]. Given these limitations, polysome profiling was developed and allows the isolation of RNA bound by multiple ribosomes that are actively translated, thus distinguishing untranslated complexes [108]. Another reliable analysis is biological MS, which can directly detect peptides encoded by ncRNAs [9]. The techniques listed above can help us identify the peptides/proteins encoded by ncRNAs, but it is necessary to conduct experimental approaches to verify whether ncRNAs are truly coding. Techniques such as western blotting, microscopy, immunohistochemistry, and CRISPR/Cas9-mediated gene editing are widely used for this purpose [109,110].

Even if several advanced technologies and bioinformatics methods have been developed, there are still some difficulties needed to be resolved. 1. The abundance of micropeptides is relatively low, and they are hard to be detected by MS in mammalian cells. 2. There are some low expressions of ncRNAs and their translated proteins/peptides in cancers, while the underlying mechanisms by which regulating the expression and stability of these peptides/proteins remains unclear. Though as it mentioned above, SHPRH-146aa was encoded by the circular form of SHPRH and protected SHPRH from degradation through UPS, which in turn increase or maintain the expression of SHPRH-146aa [74]; In addition, Guo et al. verified that activation of TGF-b/Smad pathway leads to increased expression of 4E-BP1, which reduced expression of CIP2A-BP through directly binding to eIF4E [38]. We still need to mine for more specific mechanisms that facilitate the discovery of new technologies. 3. Ivanov et al. reported the formation of circRNA relied on the RNA-editing enzyme ADAR [111]. And ADARs-mediated circRNA regulation exhibited in multiple cancer types, including HCC, GBM and CRC [112]. Besides, ADAR2 mediated-RNA editing reduced the stability of Alu elements and inhibited the formation of circRNAs [113]. What’s more, A to I Alu RNA editing regulated the stability of lncRNA NEAT1 in cardiovascular disease [114]. Therefore, RNA editing had a great influence on the formation and expression of ncRNAs, which also could affect the expression of peptide from the source. This suggested that RNA editing may be an initiating mechanism to drive translation. 4. Western blotting and immunohistochemistry are less sensitive in the detection of antibodies produced by putative peptides; Therefore, we are expecting more advanced detection methods and experimental technologies to appear in the future.

### 4.2. Outlook for Clinical Applications

Early diagnosis and proper treatments of cancers have meaningful impacts on tumor development and prognosis. As listed in Table 1, the levels of these peptides/proteins are increased/decreased in diverse cancers and are associated with some important clinical characteristics, such as clinical stage, lymphatic metastasis, overall survival (OS) and disease-free survival (DFS). Hence, they may act as essential biomarkers in the early diagnosis, prognostic determination and monitoring of recurrence in cancers. Conventional serum tumor biomarkers, such as CEA, CA125, and CA199, are recommended for cancer detection due to their specificity and sensitivity in the early stages of cancer [115]. Li et al. found high-frequency detection of translatable circARHGAP35 in 35 blood extracellular vesicle (EV) samples from HCC patients, which is useful for identifying peptides/proteins as tumor markers in the future [18].

In recent decades, a large number of antitumor drugs have emerged, including small molecule targeting drugs related to signaling pathways, antiangiogenic drugs, ubiquitin-proteasome inhibitors, monoclonal antibodies, gene therapy, et al. As we have summarized above, peptides/proteins play stimulative/suppressive roles through a variety of different pathways, and they can be considered as therapeutic targets in cancer treatment. For instance, TNBC is known as an invasive disease of estrogen receptor (ER)(-), progesterone receptor (PR)(-), and HER(-), with a high risk of distant metastasis, and often occurs in young women [116]. Pertuzumab is routinely used in HER2-positive patients by inhibiting HER2 heterodimerization [117]. Li et al. reported that HER2-103 translated from circHER2 shared the same antigen-recognition domain of HER2 that could be antagonized by pertuzumab, and in vivo studies showed that pertuzumab attenuated the tumorigenicity of circHER2/HER2-103-positive TNBC cells [39]. This finding suggests that we can try to screen TNBC patients expressing circHER2/HER2-103 in future clinical trials to verify the effect of pertuzumab.

Moreover, cancer vaccines have received much attention in recent years. The advantage of cancer vaccines is that they can produce long-term immune memory and have a relatively lasting antitumor effect. Several cancer vaccines are available in clinical therapy, including Melacine for melanoma and Cima Vax EGF for lung cancer [118,119]. Céline M. Laumont et al. pointed out that tumor-specific antigens (TSAs) are desired targets for immunotherapy and found that most TSAs detected in human primary tumors were generated from the translation of noncoding regions. Moreover, the evaluation of the efficacy of TSA vaccination in mice suggested that immunization with individual TSAs provides varying degrees of protection to EL4 cells, and this protection is long lasting. Accordingly, TSAs from noncoding regions can be a promising approach in cancer immunotherapy [120]. With intensive research, an increasing number of ncRNA-encoded proteins/peptides have been discovered, and we are expecting that these discoveries can ultimately be applied to further clinical investigations.

## 5. Conclusions

So far, we’ve cracked the mystery of translatable ncRNAs, however, studies on functions and mechanisms of ncRNA-encoded peptides/proteins are still in its infancy. Firstly, thousands of ORFs have been found in different species, so are there more translation mechanisms of ORFs? Secondly, though there are already many technologies that excavate unknown peptides/proteins encoded by ncRNAs, these technologies have certain limitations, and we need to overcome the detection obstacles and optimize the technologies. Thirdly, research of ncRNA-encoded peptides/proteins are concentrated on cell and animal levels, more human samples should be considered to bring into the research, like blood samples. Finally, ncRNA-encoded peptides/proteins have biological and regulatory functions, and analysis of their network with other pathogenic genes in tumorigenesis is of great significance for the development of anticancer drugs.

## Figures and Tables

**Table 1:** Levels and clinical significances of ncRNAs-encoded peptides/proteins in cancers. ↑ means high expression; ↓ means low expression.

Cancers	ncRNAs	Peptides/Proteins	Peptides/Proteins Expression	Clinical Significance of Peptides/Proteins
HCC	circβ-catenin	β-Catenin-370aa	↑	-
HCC	LINC00998	SMIM30	↑	advanced clinical stage; shorter OS and DFS
HCC	lncRNA HBVPTPAP	HBVPTPAP	↓	-
HCC	circPINT	PINT87aa	↓	-
HCC	Circ ARHGAP35	circ AHGAP3 protein	↑	-
GBM	circular AKT3	AKT3-174aa	↓	longer OS
GBM	circular SMO	SMO-193aa	↑	-
GBM	circ-FBXW7	FBXW7-185aa	↓	-
GBM	circSHPRH	SHPRH-146aa	↓	longer survival time
GBM	circ-EGFR	rtEGFR	↑	-
GBM	circPINT	PINT87aa	↓	earlier clinical stage
CRC	lncRNA HOXB-AS3	HOXB-AS3 peptide	↓	less metastasis; earlier clinical stage; lower risk of cancer death; longer OS
CRC	circMAPK14	circMAPK14-175aa	↓	-
CRC	circPLCE1	circPLCE1-411	↓	-
CRC	LINC00266-1	RBRP	↑	highly metastasis; advanced clinical stage; higher risk of cancer death; shorter OS
CRC	circ_0006401	circ_0006401 peptide	↑	highly lymphatic metastasis
CRC	lncRNA LOC90024	SRSP	↑	advanced clinical stage; higher risk of cancer death; shorter OS; shorter median survival time
CRC	linc00675	FORCP	↓	well differentiated cancer
CRC	lincPVT1	PVT1 peptide	↑	-
CC	circPPP1R12A	circPPP1R12A-73aa	↑	-
CC	circFNDC3B	circFNDC3B-218aa	↓	-
GC	circAXIN1	AXIN1-295aa	↑	-
GC	circMAPK1	MAPK1–109aa	↓	longer OS
GC	CircDIDO1	DIDO1-529aa	↓	-
TNBC	LINC00665	Micropeptide CIP2A-BP	↓	longer OS
TNBC	circ-HER2	HER2–103	↑	-
TNBC	circFBXW7	FBXW7-185aa	↓	-
TNBC	circ-EIF6	EIF6-224aa	↑	-
TNBC	LINC00908	ASRPS	↓	longer OS
gefitinib resistance in LAUD	circASK1	ASK1-272aa	↓	shorter PFS
bladder cancer	circGprc5a	Gprc5a	↑	-
neuroblastoma	circCUX1	p113	↑	-
AML	ASH1L-AS1	APPLE	↑	-
endometrial cancer	circ-0000437	CORO1C-47aa	↓	-
cervical cancer	circE7	E7 oncoprotein	↑	-
